# Open‐label study with the monoamine stabilizer (‐)‐OSU6162 in myalgic encephalomyelitis/chronic fatigue syndrome

**DOI:** 10.1002/brb3.2040

**Published:** 2021-02-02

**Authors:** Sara Haghighi, Sara Forsmark, Olof Zachrisson, Arvid Carlsson, Marie K. L. Nilsson, Maria L. Carlsson, Robert C. Schuit, Carl‐Gerhard Gottfries

**Affiliations:** ^1^ Department of Neurology Motala Hospital Motala Sweden; ^2^ Gottfries Clinic Affiliated with Institute of Neuroscience and Physiology The Sahlgrenska Academy University of Gothenburg Gothenburg Sweden; ^3^ Department of Clinical Neuroscience Institute of Neuroscience and Physiology The Sahlgrenska Academy University of Gothenburg Gothenburg Sweden; ^4^ Amsterdam University Medical Center VU University Medical Center Amsterdam The Netherlands

**Keywords:** fatigue, ME/CFS, monoaminergic stabilizer, mood

## Abstract

**Objectives:**

The purpose of the present study was to investigate the safety and tolerability of the monoaminergic stabilizer (‐)‐OSU6162 in patients with myalgic encephalomyelitis/chronic fatigue syndrome (ME/CFS). In addition, a potential therapeutic effect of (‐)‐OSU6162 in ME/CFS was evaluated by means of observer‐rated scales and self‐assessment rating scales.

**Materials and Methods:**

In the current study using an open‐label single‐arm design ME/CFS patient received treatment with (‐)‐OSU6162 during 12 weeks. The patients received the following doses of (‐)‐OSU6162: 15 mg b.i.d. during the first 4‐week period, up to 30 mg b.i.d. during the second 4‐week period and up to 45 mg b.i.d. during the third 4‐week period, with follow‐up visits after 16 and 20 weeks.

**Results:**

Out of 33 included patients, 28 completed the 12 weeks treatment period. (‐)‐OSU6162 was well tolerated; only one patient discontinued due to an adverse event. Vital signs and physical examinations showed no abnormal changes. Blood analyses showed an increase in serum prolactin. Therapeutically, improvements were seen on the Clinical Global Impression of Change scale, the FibroFatigue scale, the Mental Fatigue Scale, the Fatigue Severity Scale, Beck Depression Inventory, and the Short Form 36 Health Survey Questionnaire.

**Conclusions:**

(‐)‐OSU6162 is well tolerated in ME/CFS patients and shows promise as a novel treatment to mitigate fatigue and improve mood and health‐related quality of life in ME/CFS. Obviously, the present results need to be confirmed in future placebo‐controlled double‐blind trials.

## Graphical abstract

We investigate the safety and tolerability of the monoaminergic stabilizer (‐)‐OSU6162 in patients with myalgic encephalomyelitis/Chronic fatigue syndrome (ME/CFS). In addition, a potential therapeutic effect of (‐)‐OSU6162 was evaluated by means of observer‐rated scales and self‐assessment rating scales. (‐)‐OSU6162 is well tolerated and improve health related quality of life in myalgic encephalomyelitis/chronic fatigue syndrome.

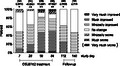

## 1 INTRODUCTION

Myalgic encephalomyelitis/chronic fatigue syndrome (ME/CFS) is a complex, multi‐system chronic neurological disorder (ICD CODE G93.3) (Bested & Marshall, 2015; Cortes Rivera et al., 2019; Zachrisson, 2002) and has a prevalence of 0.1%–6.4% (Brurberg et al., 2014; Nacul et al., 2011; Sharpe et al., 1991).

Apart from pathological fatigue which is the dominating symptom in ME/CFS patients suffer from postexertional malaise, pain, sleep disturbance, and neurocognitive dysfunctions like impaired short‐term memory and reaction time, and concentration difficulties (Bested & Marshall, 2015; Cortes Rivera et al., 2019; Hardcastle et al., 2016). Dysfunctions in the immune, neuroendocrine and autonomic nervous system are also common. The cause of ME/CFS is still unknown and treatment is limited to symptom relief (Blomberg et al., 2018; Moneghetti et al., 2018; Schutzer et al., 2011).

(‐)‐OSU6162 has in preclinical studies been shown to stabilize brain dopaminergic and serotonergic signaling (Carlsson et al., 2011). In short‐term double‐blind studies, with maximally four weeks’ exposure to active treatment, (‐)‐OSU6162 has shown a favorable safety and tolerability profile and, in addition, promising therapeutic effects (Berginstrom et al., 2019; Johansson et al., 2012; Khemiri et al., 2015; Kloberg et al., 2014; Nilsson et al., 2018).

This study is a part of a larger open‐label study investigating the safety and tolerability of (‐)‐OSU6162 in patients suffering from mental fatigue and related vitality and alertness disturbances in different neurological disorders following treatment during a more extended time period (12 weeks) compared to previous studies (maximally four weeks). From this larger study we have earlier reported on open administration of (‐)‐OSU6162 in multiple sclerosis (Haghighi et al., 2018). In the present part of the study, we investigated the safety, tolerability and potential therapeutic effects of (‐)‐OSU6162 in ME/CFS patients.

## 2 MATERIALS AND METHODS

### 2.1 Patients

Patients diagnosed with ME/CFS according to the Fukuda (Fukuda et al., 1994) and the International Consensus Criteria (ICC; Carruthers et al., 2011) were recruited from the Gottfries Clinic AB, Mölndal, Sweden, a Clinic specialized in ME/CFC and fibromyalgia. The study was carried out at Gottfries Clinic.

The diagnostic criteria for ME/CFS are:

- Pathological fatigue, postexertional malaise, sleep problems, pain, two neurocognitive symptoms, and at least one symptom from two of the following categories: autonomic nervous system, endocrine system, immune system.
- The fatigue and the other symptoms must persist or be relapsing for at least 6 months. A provisional diagnosis may be possible earlier.
- The symptoms cannot be explained by another illness (Friedberg et al., 2014).

The patients had to be between 18 and 75 years old and be essentially healthy apart from ME/CFS. Laboratory samples were taken at the screening visit, prior to inclusion, to exclude other causes of fatigue (e.g., anemia, thyroid disorder, vitamin B12 / folate deficiency, inflammation / infection). The patients had previously been somatically investigated at Gottfries Clinic and subjected to thorough interviews and routine laboratory analyses to exclude other disorders. Additionally, at the screening visit patients underwent physical examination, check of vital signs, blood sampling for analyses specified below in *Safety evaluation*, ECG, UCG as well as drug and pregnancy tests. Patients who showed pathological abnormalities on ECG and UCG, and patients with clinically significant abnormal laboratory values were not allowed to participate in the study. Other important exclusion criteria for participation in the present study were other serious somatic or psychiatric disease including severe depression (Beck Depression Inventory score ≥ 30), alcohol/drug abuse, women of childbearing age not taking contraceptives, pregnant or breastfeeding women.

Co‐morbidity in the form of fibromyalgia and irritable bowel syndrome (IBS) did not exclude participation. Previous participation in clinical studies with (‐)‐OSU6162 was allowed (23 of the included patients had about two years earlier participated in a clinical study with the study drug, seven of them were exposed to OSU6162).

Certain concomitant medications, for example, antidepressants, anxiolytics and hypnotics, were allowed if they were kept stable during the 3 months preceding study start and throughout the study.

Before entering the study, patients gave their informed consent after receiving information about the aims, methodology, potential risks and anticipated benefits of the study.

Patients could terminate their participation in the present study whenever they wished and they were not required to provide an explanation for their withdrawal.

### 2.2 Procedure

**FIGURE 1:**
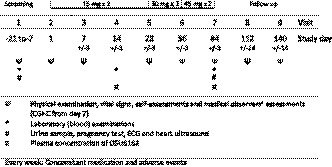
Study flow chart

In this open‐label single‐arm study, we used an individualized, flexible, stepwise increasing (‐)‐OSU6162 dosing procedure; if a scheduled dose increase resulted in decreased therapeutic effect and/or adverse event(s), the lower dose would be resumed and could be the final dose for that patient. The treatment period lasted 84 days (12 weeks) with follow‐up visits at day 112 and 140. The patients received the following doses of (‐)‐OSU6162: 15 mg b.i.d. during the first 28 days, up to 30 mg b.i.d. during the following 28 days and up to 45 mg b.i.d. during the last 28 days. The (‐)‐OSU6162 tablets were taken in the morning and at noon. A study flow chart is shown in Figure 1.

Samples for measurements of (‐)‐OSU6162 plasma concentrations were drawn on all patients at day 14 and 84 about 90 min after medication intake in the morning. Plasma concentrations of (‐)‐OSU6162 were determined by high‐performance liquid chromatography/tandem mass spectrometry as described previously (Tolboom et al., 2015).

### 2.3 Safety evaluation

Safety evaluation included registration of adverse events (AEs), vital signs (blood pressure, pulse rate and weight), physical examination as well as electrocardiography (ECG), heart ultra sound (UCG) and blood samples analyzed for concentration of hemoglobin, leukocytes, thrombocytes, C‐reactive protein, sodium, potassium, creatinine, calcium, aspartate aminotransferase, alanine aminotransferase, alkaline phosphatase, albumin, bilirubin, gamma‐glutamyl transferase, thyroid‐stimulating hormone, free thyroxine, prolactin, and for determination of erythrocyte sedimentation rate. Urine samples were analyzed for content of glucose and protein and were also tested for drug abuse and pregnancy. Time schedule for measurements is shown in Figure 1.

### 2.4 Efficacy evaluation

#### 2.4.1 Medical observers' rating scales

Clinical Global Impression of Change (CGI‐C; Guy, 1976) is a 7‐point scale where a skilled and experienced clinician makes an assessment of how much the participant's illness has improved or worsened relative to a baseline state at the beginning of the study and it is rated as: 1 = very much improved; 2 = much improved; 3 = minimally improved; 4 = no change; 5 = minimally worse; 6 = much worse; 7 = very much worse.

The FibroFatigue scale (FF), specifically constructed for measuring symptom severity and treatment outcome in fibromyalgia and chronic fatigue syndrome patients (Zachrisson et al., 2002), consists of 12 observer‐rated items. The scale is validated and contains in addition to questions related to fatigue also questions about, for example pain, muscular tension, headache and infection feelings. Structured interviews with participants are the basis for the skilled and experienced clinician´s scoring on a 7‐point scale ranging from 0 to 6, where higher scores reflect more symptoms. The scores from the 12 items are summarized into a total score (0–72).

#### 2.4.2 Self‐rating scales

The Mental Fatigue Scale (MFS; Johansson et al., 2010) is a questionnaire consisting of 15 items with focus on mental aspects of fatigue. The scale covers sleep, sensory, emotional and cognitive domains, mental recovery and diurnal variation. The 15 items were summarized into a total score; more severe symptoms are reflected in higher total scores. Range of total scores 0–44. Suggested evaluation of scores: 0–10, normal, 10,5–14,5 mild, 15–20 moderate, >20 severe symptoms of mental fatigue. This scale has in our previous studies with (‐)‐OSU6162 shown high sensitivity with respect to the mental fatigue symptomatology and was included here for, for example, comparative purposes.

The Fatigue Severity Scale (FSS; Krupp et al., 1989) is a 9‐item validated scale that measures the severity of fatigue in relation to physical and other activities. The items are scored on a 7‐point scale with 1 = strongly disagree and 7 = strongly agree; more fatigue results in higher scores. The scores from the 9 items were summarized into a total score. This scale was required by the Swedish Medical Products Agency.

The Beck Depression Inventory (BDI; Beck et al., 2005) consists of 21 items concerning symptoms/attitudes assessed on a 4‐point scale ranging from 0 to 3. By summarizing the scores of the 21 items, a total score is obtained; the more severe symptoms, the higher total scores. Range of total scores 0–63. Suggested interpretation of scores 0–13 minimal, 14–19 mild, 20–28 moderate, 29–63 severe symptoms of depression.

Visual Analog Scale (VAS) was used for assessment of pain. On a 10 cm long, horizontal line marked with “no pain” and “worst possible pain” in respective end, patients were asked to mark the point along the line that most accurately expressed her/his degree of pain.

The Short Form 36 Health Survey Questionnaire (SF‐36; Sullivan, 2002) is a 36‐item survey consisting of eight scaled scores, which are the weighted sums of the questions in each section. Each scale is directly transformed into a 0–100 scale on the assumption that the questions are equally important; a higher degree of disability results in a lower score. The eight sections are vitality, social function, role emotional, mental health, physical function, role physical, bodily pain and general health. The first four and the last four sections are summarized into a mental and a physical health component, respectively.

CGI‐C was carried out at day 7, 28, 56, 84, 112, and 140.

FF, MFS, FSS, BDI, VAS and SF‐36 were carried out at screening, inclusion (day 1), day 7, 28, 56, 84, 112, and 140.

### 2.5 Ethics

The study was conducted in agreement with the declaration of Helsinki (64th WMA General Assembly, Fortaleza, Brazil, October 2013) and with international conference on Harmonization and guidelines on Good Clinical Practice. The Göteborg Medical Ethics Committee gave their approval to the study (Dnr 852‐13). Eudra Nr 2013‐002545‐10.

### 2.6 Statistics

Analyses of change over time were performed with Fisher's nonparametric permutation test for paired observations (Good, 2000). For construction of 95% confidence intervals for the mean change, bootstrapping with 10,000 replicates was used. Correlations were performed with Spearman rank correlation. All efficacy analyses were subjected to Bonferroni–Holm correction for multiple comparisons (16 efficacy comparisons at each time point); both adjusted and unadjusted *p*‐values are presented. Safety analyses are given with unadjusted *p*‐values. Analyses were performed on existing data; thus, no imputing techniques were applied for missing data. All tests were two‐tailed. Analyses were performed with SAS^®^ v9.4.

## 3 RESULTS

Out of 38 patients screened, 33 fulfilled the inclusion criteria and were included in the study. Two patients terminated early after visit 3 due to tiredness and lack of motivation. Another patient withdrew after visit 6 due to an adverse event (affect lability). Further, five patients dropped out at their own discretion after visit 5 (one patient), 6 (one patient) and 7 (day 84, after end of treatment; three patients) without giving any reason for drop out; thus, 28 patients completed treatment to day 84. Twenty‐five completed to first follow‐up and 15 patients completed to second follow‐up. Demographics and baseline assessments are shown in Table 1. For the participants who completed the study final daily doses were 15 (*n* = 2), 30 (*n* = 8), 45 (*n* = 2), 60 (*n* = 6), 75 (3) and 90 mg (*n* = 4), that is, a mean final daily dose of 52.2 mg.

**TABLE 1:** Demographics. Shown are *n* (%) or mean (*SD*)/median (min; max)

	(*n* = 33)
Gender	
Male	6 (18.2%)
Female	27 (81.8%)
Ethnic group	
Caucasian	33 (100%)
Smoking	1 (3.0%)
Other nicotine use	3 (9.1%)
Age at inclusion, years	49.8 (11.4)/ 50.0 (25.2; 71.2)
Years since diagnosis	5.4 (5.2)/ 4.0 (0.6; 18)
Weight (kg)	72.7 (13.0)/ 68.3 (51.4; 106.0)
Height (cm)	169.2 (8.3)/ 169.0 (152.0; 192.0)

Note

Shown are *n* (%) or mean (*SD*)/median (min; max).

### 3.1 Safety evaluation

Adverse events are summarized in Table 2. Two participants experienced serious adverse events (SAEs) during the study: One participant was afflicted with appendicitis and one participant with bronchopneumonia who later on also suffered from anxiety (leading to hospitalization); none of these SAEs were judged to be related to treatment. One patient discontinued due to an adverse event (affect lability) with start during the second month of treatment (labelled as related to treatment with mild intensity). In general, adverse events were predominantly of mild intensity and decreased in number during the course of the study. See Table 2 for the most common AEs reported.

**TABLE 2:** Summary of adverse events. Shown are total number of SAEs, AEs and number of subjects reporting at least one AE, *n* (%) and the most common reported AEs by preferred term

	Total (*n* = 33)		During 1st month (*n* = 33)		During 2nd month (*n* = 31)		During 3rd month (*n* = 30)		During Follow‐up period (*n* = 29)	
	AEs	Subjects	AEs	Subjects	AEs	Subjects	AEs	Subjects	AEs	Subjects
Any SAE	3	2 (6.1%)	1	1 (3.0%)	0	0 (0.0%)	2	2 (6.7%)	0	0 (0.0%)
Any AE	160	31 (93.9%)	76	27 (81.8%)	37	20 (64.5%)	31	12 (40.0%)	16	11 (37.9%)
Maximum reported intensity										
Mild	115	29 (87.9%)	62	23 (69.7%)	29	17 (54.8%)	15	7 (23.3%)	9	8 (27.6%)
Moderate	38	16 (48.5%)	13	9 (27.3%)	8	5 (16.1%)	11	5 (16.7%)	6	3 (10.3%)
Severe	7	5 (15.2%)	1	1 (3.0%)	0	0 (0.0%)	5	4 (13.3%)	1	1 (3.4%)
Any treatment related AE										
Yes	91	26 (78.8%)	37	18 (54.5%)	23	14 (45.2%)	23	7 (23.3%)	8	5 (17.2%)
No	69	26 (78.8%)	39	18 (54.5%)	14	10 (32.3%)	8	6 (20.0%)	8	7 (24.1%)
Most common AEs by Preferred Term^a^										
Dizziness		13 (39.4%)		9 (27.3%)		2 (6.5%)		5 (16.7%)		1 (3.4%)
Insomnia		13 (39.4%)		5 (15.2%)		7 (22.6%)		2 (6.7%)		
Nausea		10 (30.3%)		5 (15.2%)		2 (6.5%)		4 (13.3%)		1 (3.4%)
Headache		9 (27.3%)		5 (15.2%)		2 (6.5%)		1 (3.3%)		2 (6.9%)
Upper respiratory tract infection		7 (21.2%)		1 (3.0%)		3 (9.7%)		1 (3.3%)		2 (6.9%)
Pyrexia		4 (12.1%)		3 (9.1%)				1 (3.3%)		1 (3.4%)
Fatigue		4 (12.1%)		2 (6.1%)				1 (3.3%)		1 (3.4%)
Diarrhea		3 (9.1%)		2 (6.1%)		1 (3.2%)		1 (3.3%)		
Abdominal discomfort		3 (9.1%)				3 (9.7%)				
Tachycardia		2 (6.1%)		2 (6.1%)						1 (3.4%)
Pain		2 (6.1%)		2 (6.1%)						1 (3.4%)

^a^ AEs reported by at least two patients.

Blood analyses revealed a slight decrease in leukocytes; from 5.88 (*SD* 0.95) to 5.44 (*SD* 1.03) x10 E9/l, *p* = .0003. One patient's leukocyte particle concentration was below reference limit at end of treatment. There was also an expected increase in S‐prolactin, from 216.4 (*SD* 95.2) to 393.2 (*SD* 234.1) mIU/L, *p* < .0001. Two prolactin values were below reference range at inclusion; 9 were above at the end of treatment, one of which was considered clinically significant (1 month after end of treatment the prolactin level had returned to normal). Vital signs and physical examinations showed no abnormal changes, and all ECG and UCG measures were normal before as well as after the 3 months of (‐)‐OSU6162 treatment.

### 3.2 Clinical efficacy evaluation

**FIGURE 2:**
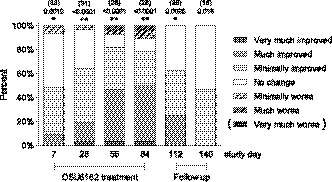
Distribution of CGI‐C scores over the study visits. The bar graph shows the percentage distribution of the CGI‐C scale's scores for each assessment point. At Day 84, 78.6% of patients were scored as improved (“Minimally improved”, “Very much improved” or “Much improved”). For comparisons within groups, the Fisher´s nonparametric permutation test for paired observations was used. Above each bar is shown number of subjects and unadjusted p‐values. Asterisks denote significance level after Bonferroni–Holm adjustment for multiple comparisons (adjusted for 16 efficacy comparison at each time point). *p* < .05*; *p* < .01**

CGI‐C scores are shown in Figure 2. Within group comparisons showed significant improvements from first assessment on day 7 and continued to be significant at day 28, 56, 84 and 112 (After Bonferroni–Holm correction: *p* < .015, *p* < .0016, *p* < .0016, *p* < .0016, and *p* < .030, respectively). On the last day (84) of (‐)‐OSU6162 treatment, 78.6% of the participants had attained different degrees of improvement (one patient (3.6%) was very much improved, 13 (46.4%) much improved, eight (28.6%) minimally improved, three (10.7%) unchanged, two (7.1%) minimally worse and one (3.6%) much worse. At the second follow‐up visit (day 140) 53.3% of the patients were scored as unchanged compared to the subjects’ baseline state at the beginning of the study.

**FIGURE 3:**
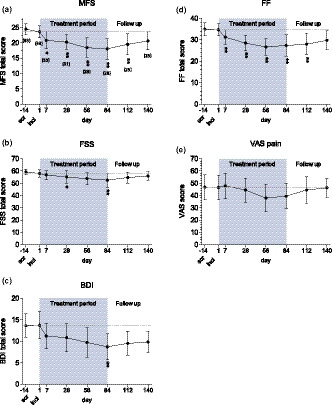
Assessments over time. Shown are mean total score and *SD* for the mean at each assessment point during the study on a) Mental Fatigue Scale (MFS) (b) Fatigue Severity Scale (FSS) (c) Beck Depression Inventory (BDI) (d) Fibro Fatigue scale and e) Visual Analog Scale for pain. The shaded area indicates start and end of treatment period. Dashed line shows mean score at inclusion. In figure (a) the number of patients is given in brackets. Scr = screening visit, Incl = inclusion visit. Asterisks denote significance level after Bonferroni–Holm adjustment (adjusted for the 16 efficacy comparisons at each time point including CGI‐C) *p* < .05*; *p* < .01**

Effects of (‐)‐OSU6162 on the efficacy variables FSS, MFS, BDI, FF, VAS and SF‐36 are shown in Table 3. There was a significant improvement in total scores of the FSS, MFS, BDI and the FF, as well as the SF‐36 sub scales Vitality, Social function and Physical function following 84 days' treatment with (‐)‐OSU6162. MFS and FF at the group level showed a reduction in mental fatigue from severe to moderate, and BDI showed a reduction of depression from mild to minimal; these changes are interpreted as clinically relevant. There were no significant changes from inclusion to last follow‐up. Figure 3 shows self‐assessments for MFS, FSS, BDI and VAS, as well as observer‐rated FF over time.

**TABLE 3:** Exploratory efficacy outcomes. Shown are mean (*SD*) and mean (95%CI) for the change. For comparison over time, a linear nonparametric permutation test for paired observations was used

	Inclusion (*n* = 33)	Change from Inclusion to Day 84 (*n* = 28)		Change from Inclusion to Day 140 (Follow‐up; *n* = 15)	
Efficacy measure	Mean (SD)	Mean (95% CI)	*p*‐value^1^	Mean (95% CI)	*p*‐value^1^
FSS total score	57.9 (6.4)	−5.29 (−8.54 to −2.42)	≤.0001**	−0.600 (−3.333 to 2.091)	.71
MFS total score	23.6 (4.0)	−5.30 (−7.26 to −3.32)	.0001**	−2.13 (−4.17 to −0.27)	.050
BDI total score	13.7 (6.3)	−4.00 (−6.18 to −2.06)	.0001**	−1.87 (−5.18 to 1.00)	.30
FF total score	34.6 (5.6)	−7.31 (−10.29 to −4.40)	≤.0001**	−4.14 (−7.00 to −1.46)	.012
VAS	46.6 (19.9)	−8.23 (−15.11 to −1.19)	.030	−1.15 (−11.59 to 9.08)	.84
SF−36:					
Component mental	39.7 (12.0)	4.24 (0.86 to 8.00)	.028	−0.477 (−4.615 to 3.798)	.82
Vitality	13.6 (15.5)	13.6 (4.5 to 22.4)	.0062*	3.67 (−6.67 to 12.50)	.52
Social function	29.2 (17.6)	18.8 (11.3 to 26.5)	≤.0001**	12.5 (3.9 to 21.3)	.015
Role emotional	60.6 (45.2)	13.1 (−1.5 to 28.7)	.11	−2.22 (−25.64 to 22.23)	1.00
Mental health	65.7 (16.9)	4.29 (−2.00 to 10.67)	.20	−2.93 (−7.33 to 1.33)	.22
Component physical	25.3 (12.3)	4.34 (1.90 to 6.93)	.0024*	3.21 (−0.10 to 6.88)	.092
physical Function	47.9 (16.9)	11.6 (5.5 to 17.9)	.0012*	6.33 (−0.48 to 13.64)	.099
Role physical	6.06 (12.55)	13.4 (3.2 to 25.8)	.030	10.00 (−3.85 to 27.50)	.38
Bodily pain	35.0 (14.9)	8.11 (2.18 to 13.90)	.014	3.73 (−4.71 to 12.42)	.41
General Health	25.4 (18.5)	3.32 (−0.23 to 7.09)	.090	−2.20 (−6.82 to 2.39)	.36

Note

Shown are mean (*SD*) and mean (95% CI) for the change. For comparison over time, a linear nonparametric permutation test for paired observations was used.

^1^ Asterisks denote significance level after Bonferroni–Holm adjustment; *p* < .05*; *p* < .01** Bonferroni–Holm adjustment was done to keep an overall constant alpha level of 0.05 at each assessment point (adjusted for 16 efficacy comparison at each assessment point including CGI‐C).

### 3.3 Relation to (‐)‐OSU6162 plasma concentration

Plasma concentrations of (‐)‐OSU6162 were determined in all patients. For those participants who completed the study (*n* = 28) the mean (‐)‐OSU6162 plasma concentration was 0.488 µM (*SD* 0.331), median 0.452 µmol/L (min; max 0.001; 1.550) on the last day of treatment. (‐)‐OSU6162 dose taken at visit 7 was significantly correlated with plasma concentration at visit 7, *r*_s_ = .82 (*p* < .0001). Likewise, (‐)‐OSU6162 plasma concentration was significantly correlated with change in serum prolactin, *r*_s_ = .51 (*p* = .0098), but not with change in blood leukocyte particle concentration. We did not detect any significant correlations between (‐)‐OSU6162 plasma concentrations and score changes observed in the clinical ratings.

## 4 DISCUSSION

In agreement with previous short‐term studies (Johansson et al., 2012; Khemiri et al., 2015; Kloberg et al., 2014; Nilsson et al., 2018) (‐)‐OSU6162 was in the present study, where the patients were exposed to (‐)‐OSU6162 for a period of 12 weeks, found to be safe and well tolerated. The adverse events were in general mild and transient or disappeared after dose reduction.

Our present observations regarding therapeutic effects on fatigue and mood in ME/CFS patients are also in accordance with earlier clinical observations. In our double‐blind crossover study in patients with enduring mental fatigue following stroke or traumatic brain injury, (‐)‐OSU6162 treatment caused an improvement on the mental fatigue scale (Johansson et al., 2012). In another double‐blind crossover study in Huntington's disease patients, we observed that OSU6162 treatment improved both the SF‐36 Vitality score and depressive symptoms rated by BDI (Kloberg et al., 2014).

Further, in a double‐blind two‐armed study of two weeks duration in ME/CFS, (‐)‐OSU6162 treatment caused mitigation of MFS‐rated fatigue in a subgroup of patients who were on concomitant pharmacological treatment for depression (Nilsson et al., 2018). Also in the present study the therapeutic response to (‐)‐OSU6162 with respect to fatigue appeared to be larger in patients receiving pharmacological treatment for depression compared to patients not receiving such treatment. Several of the patients in the present study reported spontaneously that they wished to continue with the (‐)‐OSU6162 treatment.

Our results also confirm previous observations (Haghighi et al., 2018) that the mental fatigue scale in the present context appears to be a more sensitive tool than the validated Fatigue Severity Scale to show clinical improvement regarding reduction of mental fatigue and related symptoms in different neurological disorders.

The reduction of mental fatigue symptoms in patients with ME/CFS and other neurological disorders after (‐)‐OSU6162 treatment may be due to the stabilizing effects of this substance on brain dopaminergic activity. Dopamine plays an important role for wakefulness and we have previously observed that (‐)‐OSU6162 stimulates behavior in habituated rats displaying a low activity level, an effect we believe is mediated by increased dopamine release resulting from dopamine autoreceptor blockade (Rung et al., 2008; Tolboom et al., 2015).

There are some potential limitations to the study: Twenty‐three of the included participants had about two years earlier participated in a clinical study with the study drug, in which 7 of them were exposed to OSU6162. This could be a potential bias which might affect both efficacy and safety data in the present study, but we could not see that the results from these patients differed from the others with respect to drop out rate, blood variables, occurrence of reported AEs or efficacy ratings. There was no tendency suggesting that those with good response to treatment in the former study were more likely to participate in the current study. The majority of patients from the former study were from the placebo group.

Further, the open design character of the current study is a limitation, future trials using a double‐blind placebo‐controlled protocol are required to show that clinical improvement is not merely due to a placebo effect. Worth mentioning in this context, though, is that experience from previous studies with ME/CFS generally shows a relatively modest placebo response, due to ME patients’ low expectations for improvement (Cho et al., 2005).

In conclusion, the results from the present 12‐week study confirm previous short‐term studies reporting that (‐)‐OSU6162 is safe and well tolerated. Further, the present and earlier findings suggest that this compound may have beneficial effects on fatigue and mood in ME/CFS and other neurological disorders.

## CONFLICTS OF INTEREST

None declared.

## AUTHOR CONTRIBUTIONS

A.C., C‐G.G., M.C., S.H., O.Z., and S.F. took part in the study design. S.H., O.Z., S.F, and C‐G.G performed the clinical evaluation. R.S. carried out the high‐performance liquid chromatography analysis. M.N performed the statistical evaluation. All authors contributed to the interpretation of data. M.N., M.C. and S.H wrote the manuscript draft, which was critically revised and finally approved by all authors.

### Peer Review

The peer review history for this article is available at https://publons.com/publon/10.1002/brb3.2040.

## Data Availability

The data that support the findings of this study are available on request from the corresponding author. The data are not publicly available due to privacy or ethical restrictions.
